# Association between health insurance membership and anaemia among children under-five years. Evidence from Ghana

**DOI:** 10.1371/journal.pone.0238792

**Published:** 2020-09-14

**Authors:** Emmanuel Anongeba Anaba, Aaron Asibi Abuosi, Joshua Cobby Azilaku, Jacqueline Nkrumah

**Affiliations:** 1 Department of Population, Family and Reproductive Health, University of Ghana, Accra, Ghana; 2 Department of Public Administration and Health Services Management, University of Ghana, Accra, Ghana; 3 Kintampo College of Health and Well-being, Kintampo, Ghana; 4 Department of Health Administration and Education, University of Education-Winneba, Winneba, Ghana; University of Georgia, UNITED STATES

## Abstract

**Introduction:**

Anaemia is prevalent among children in developing countries. The main objective of this study was to assess the association between health insurance membership and anaemia among Ghanaian children under-five years.

**Methods:**

We obtained Ghana’s Multiple Indicators Cluster Survey, 2011 dataset from the United Nations International Children’s Emergency Fund. Data were analyzed with the aid of Stata/IC, version 15.

**Results:**

The prevalence of anaemia among Ghanaian children under-five years was estimated to be 57%. Majority (73%) of the children were not insured. Health insurance membership was found to be a significant predictor of anaemia among children under-five years.

**Conclusion:**

Health insurance membership is a protective factor against anaemia among children under-five years. In the quest to eradicate anaemia among children, stakeholders would have to review the benefit package of the National Health Insurance Scheme coupled with prioritizing anaemia prevention interventions among more vulnerable children.

## Introduction

Anaemia is a global public health problem. Anaemia is prevalent among children under-five years, especially in developing countries [1, 2]. In this study, anaemia refers to haemoglobin levels below or equal to10.9 g/dl. Anaemia in children is mainly caused by iron deficiency and other nutritional deficiencies, such as folate, Vitamin A and B_12_ [3]. Some of the symptoms of anaemia include dizziness, paleness of skin, eyes and palms and body tension [4]. Anaemia in children can lead to cognitive, physical and social impairments. These impairments can affect academic performance and work capacity in adulthood [5]. In addition, anaemia can lead to low immunity and growth weight, fatigue, difficulty with concentration, lethargy, increase susceptibility to infection and death [6, 7].

Globally, about 273 million of children under-five years are anaemic. Majority (60.2%) of these children live in Africa. Anaemia is one of the leading causes of under-five mortality in Africa [8, 9]. For example, a study in the coastal part of Tanzania estimated a prevalence rate of 74% [10], whilst in southern Cameroon, a prevalence rate of 45% was estimated [11]. Similar prevalence rates have been found in Ghana. For instance, the Ghana Demographic and Health Survey reported a prevalence rate of 76% and 78% in 2003 and 2008 respectively [12]. According to Ghana Health Service, anaemia is the third leading cause of hospital admission, and the fourth leading cause of death among children under-five years [13]. In terms of regional variations, anaemia is more prevalent in the Northern Region (82%) but less prevalent is the Ashanti Region (54%) [12].

Despite the implementation of anaemia prevention interventions, such as the promotion of exclusive breastfeeding, the prevalence rate of anaemia among children under-five years in Ghana (78%) is higher than the global prevalence rate of 47.4% [14]. This can derail Ghana’s effort towards achieving the Sustainable Development Goals, especially Goal three (i.e. to ensure healthy lives and promote wellbeing for all at all ages, including children) [15]. Promoting access to child healthcare services is crucial for enhancing children health and well-being [16]. Health insurance is a major intervention that promotes financial access to healthcare and reduces catastrophic healthcare expenditure [17, 18].

However, literature on health insurance and child health outcomes are mixed. For example, studies conducted in China and Costa Rica found no significant relationship between health insurance membership and child health outcomes [19, 20]. On the contrary, [21] found that household enrollment in health insurance was significantly associated with 5.7% reduction in child stunting. In addition, studies conducted in the United States of America found that the State Children’s Health Insurance Program brought improvement in child health outcomes [22, 23]. Furthermore, evidence from Taiwan shows that health insurance reduces the incidence of death among children [24].

In Ghana, there is limited literature on health insurance membership and anaemia, especially among children under-five years. Ghana is one of the countries in sub-Saharan Africa with a National Health Insurance Scheme (NHIS) [25]. Ghana’s NHIS was launched in 2005 to reduce demand side barriers to accessing healthcare. Although the impact of Ghana’s NHIS on access and utilization of maternal healthcare services have been well documented, little is known about its impact on anaemia among children under-five years [26]. Prior studies focused on health insurance membership and stunting and under-five mortality [27, 28]. The main aim of this study was to examine the association between health insurance membership and anaemia among children under-five years, using 2011 Ghana Multiple Indicators Cluster Survey data. Studies of this nature can inform child healthcare policies and interventions in Ghana.

## Materials and methods

### Data source

Data used in this study was obtained from MICS 2011, which is nationally represented. The survey provides estimates of all key health indicators at national and regional levels, as well as for urban and rural areas. MICS 2011 was conducted six years after the implementation of the NHIS in Ghana, hence, it is capable of detecting any association.

### Study design

MICS 2011 adopted a complex survey design using two-stage random sample technique [29]. First, 810 enumeration areas or clusters were selected from the 2010 Population and Housing Census list, using systematic probability proportional to size. Second, 12,150 households were selected from the enumeration areas. Third, 15 households in each cluster were selected using systematic random sampling technique. Twenty strata comprising of 10 urban and 10 rural areas were selected across Ghana. In all, 7,550 caregivers/mothers of children under-five years were interviewed. Four regions including Central, Northern, Upper East and Upper West were over sampled [29].

### Measurement

A battery-operated portable HemoCuec 201+ photometer, which reads results within a minute, was used to screen children for anaemia. The screening was done using a microcuvette from a drop of blood taken from a finger or heel prick. Results were communicated to mothers/caregivers through verbal or writing words. In addition, a brochure explaining the causes and prevention of anaemia was given to every household with a child under-five years. Details about MICS 2011 is provided elsewhere [29].

### Variable definition

In this study, the main exposure variable was health insurance membership, which is defined as a child with a valid NHIS card which was seen during the survey. The main outcome of interest was anaemia status among children under-five years. Children who had haemoglobin levels below or equal to10.9 g/dl were considered as being anaemic. Since, there was no anaemia status in the dataset, we created a new variable called ‘anaemia status’ by categorizing haemoglobin levels into ≤ 10.9 g/dl as ‘anaemic’ and ≥11g/dl as ‘not anaemic’. In addition, religion was recoded into four categories. The confounders identified in literature were socio-economic status, mother’s education, sex, age, religion, region and residential status. The MICS 2011 dataset was obtained from United Nations International Children’s Emergency Fund through a formal request. The MICS 2011 had the approval of Ghana Health Service Ethical Review Board.

### Statistical analysis

We used graphical techniques such as scatter plot to check for the possibility of outliers in the data. Since complex survey design effect is essential in estimating the precision of survey estimates, anaemia weight was applied. First, we generated a new variable called ‘weight’ by dividing anaemia weight by 1,000,000. Second, we declared survey design for dataset using the primary sampling unit, strata and the new ‘weight’. We computed descriptive statistics using frequencies and percentages. Chi-square analysis was computed to determine the association between health insurance membership, sociodemographic characteristics and anaemia among children under-five years. In addition, we computed Binary Logistic Regression, controlling for confounding factors, such as mother’s education and child’s age, to determine the significant predictors of anaemia. Since the main objective of the study was to assess the association between health insurance membership (main exposure) and anaemia (main outcome), adjusting for several independent variables, effect modification was not explored. Results were reported at the 0.05 significance level. Data were analyzed using Stata/IC, version 15 (StataCorp, College Station, Texas, USA).

## Results

### Socio-demographic characteristics of respondents

It was found that more than half (57%) of the children were anaemic. Seven in ten (73%) children were not members of the NHIS. The study also found that half of the children were females and 60.4% were above two years. Moreover, 67.5% of the mothers/caregivers had primary education and 56.5% lived in rural areas. In terms of socio-economic status, 64.1% of the mothers/caregivers were of the middle class or below. Details are provided in Table 1.

**Table 1:** Socio-demographic characteristic, anaemia and health insurance membership of children under-five years in Ghana, 2011.

Characteristic	Frequency (n)	Percentage (%)
**Sex**		
Male	3757	49.8
Female	3793	50.2
**Child’s age (months)**		
0–11	1543	20.4
12–23	1453	19.2
24–35	1553	20.6
36–47	1576	20.9
48–59	1426	18.9
**Mother’s education**		
None	2455	32.5
Primary	1628	21.6
Junior High School	2578	34.1
Senior High School and above	889	11.8
**Socio-economic status**		
Poorest	1730	22.9
Second	1551	20.5
Middle	1559	20.7
Fourth	1397	18.5
Richest	1313	17.4
**Residence**		
Urban	3283	43.5
Rural	4267	56.5
**Religion**		
Christianity	4910	65.0
Muslim	1495	19.8
Other religions (i.e. traditionalist)	595	7.9
No religion	550	7.3
**Region**		
Western	758	10.0
Central	740	9.8
Greater Accra	1142	15.0
Volta	601	8.0
Eastern	827	11.0
Ashanti	1411	18.7
Brong- Ahafo	671	8.9
Northern	852	11.3
Upper East	325	4.3
Upper West	223	3.0
**Health insurance membership**		
No	5508	73.0
Yes	2042	27.0
**Anaemia status**		
Not anaemic	1942	43.0
Anaemic	2575	57.0

### Association between health insurance membership, socio-demographic characteristics and anaemia status

We found a significant association between health insurance membership and anaemia status among Ghanaian children under-five years. It was also found that 60.6% of the anaemic children were not insured with the NHIS. We found that 75.1% of the anaemic children were of the poorest wealth quintile. In addition, anaemia was more prevalent among children under-two years. Males had a higher prevalence than females. The Upper East, Upper West and Northern Regions had prevalence of 77.5%, 81.5% and 81.2% respectively compared to 44% in the Ashanti Region. Other socio-demographic factors, such as mother’s education, residential status and age of the child were significantly association with anaemia status. Details are provided in Table 2.

**Table 2:** Cross-tabulation of socio-demographic characteristics, health insurance membership and anaemia among children-under five years in Ghana, 2011.

Characteristic	n	Not anaemic	Anaemic	Chi-square	p-value
**Health insurance membership**				31.324	< .001
No	3211	39.4	60.6		
Yes	1306	51.9	48.1		
**Socio-economic status**				40.795	< .001
Poorest	1032	24.9	75.1		
Second	944	36.0	64.0		
Middle	920	43.5	56.5		
Fourth	877	53.1	46.9		
Richest	745	64.4	35.6		
**Sex**				13.396	.003
Male	2224	39.4	60.6		
Female	2292	46.5	53.5		
**Mother’s education**				34.327	< .001
None	1493	30.5	69.5		
Primary	977	40.8	59.2		
Junior High School	1540	51.1	48.9		
Senior High School and above	507	59.3	40.7		
**Region**				17.333	< .001
Western	458	43.5	56.5		
Central	455	42.8	57.2		
Greater Accra	683	52.2	47.8		
Volta	364	41.9	58.1		
Eastern	470	53.8	46.2		
Ashanti	830	56.0	44.0		
Brong-Ahafo	400	36.7	63.3		
Northern	508	18.8	81.2		
Upper East	195	22.5	77.5		
Upper West	135	18.5	81.5		
**Residence**				40.795	< .001
Urban	1979	52.3	47.7		
Rural	2538	35.8	64.2		
**Religion**				28.023	< .001
Christian	2932	49.4	50.6		
Muslim	903	30.1	69.9		
Traditional	347	26.5	73.6		
No religion	336	39.1	60.9		
**Child’s age (months)**				16.803	< .001
0–11	482	35.8	64.2		
12–23	970	30.8	69.2		
24–35	1033	44.2	55.8		
36–47	1063	47.5	52.5		
48–59	969	52.6	47.4		

### Predictors of anaemia among children under-five years

The highest bivariate correlation coefficient between the independent variables was 0.59, which is less than the recommended threshold of > 0.70 [30], hence there was no problem of multicollinearity. The Goodness Of Fit (GOF) test showed that the model was well fitted (p-value > 0.56) [31]. In the crude analysis, it was found that health insurance membership, sex, mother’s education, residential status, socio-economic status, age, religion, and region of residence were significant predictors of anaemia status. Controlling for confounding variables, such as socio-economic status and mother’s education, health insurance membership emerged as a significant predictor of anaemia status among children under-five years. Children who were insured with the NHIS had lesser odds (AOR = .66; 95% CI: .54-.80) of being anaemic compared to children who were not insured with the NHIS. Moreover, the odds of a child of the richest wealth quintile (AOR = .35; 95% CI: .23-.54) developing anaemia was less than a child of the poorest wealth quintile. Details are provided in Table 3.

**Table 3:** Binary logistic regression of predictors of anaemia among children under-five years in Ghana, 2011.

Covariate/exposure	Crude analysis OR (95% CI)	Wald p-value	Adjusted analysis OR (95% CI)	Adjusted Wald p-value
**Health insurance membership (main exposure)**				
No	1(reference category)	< .001	1(reference category)	< .001
Yes	.60(.50-.72)		.66(.54-.80)	
**Sex**				
Male	1(reference category)	.003	1(reference category)	.002
Female	.75(.64-.87)		1.04(.79–1.36)	
**Child’s age (months)**				
0–11	1(reference category)	< .001	1(reference category)	< .001
12–23	1.25(.93–1.68)		1.24(.89–1.71)	
24–35	.70 (.52-.96)		.69(.49-.96)	
36–47	.61(.46-.82)		.53(.39-.72)	
48–59	.50(.36-.69)		.44(.31-.61)	
**Mother’s education**				
None	1(reference category)	< .001	1(reference category)	.592
Primary	.64(.51-.77)		1.04(.79–1.36)	
Junior High School	.42(.34-.51)		.89(.69–1.16)	
Senior High School and above	.30(.22-.41)		.86(.58–1.27)	
**Socio-economic status**				
Poorest	1(reference category)	< .001	1(reference category)	< .001
Second	.59(.47-.75)		.86(.65–1.14)	
Middle	.43(.32-.57)		.78(.54–1.12)	
Fourth	.29(.22-.39)		.53(.36-.79)	
Richest	.18(.14-.25)		.35(.23-.54)	
**Religion**				
Christianity	1(reference category)	< .001	1(reference category)	.023
Islam	2.26(1.79–2.84)		1.49(1.12–1.99)	
Other religions (i.e. traditionalist)	2.71(2.04–3.61)		1.35(.96–1.84)	
No religion	1.52(1.09–2.10)		1.25(.90–1.73)	
**Residence**				
Urban	1(reference category)	< .001	1(reference category)	.189
Rural	1.97(1.63–2.38)		1.18(.92–1.52)	
**Region**				
Western	1(reference category)	< .001	1(reference category)	< .001
Central	1.03(.72–1.48)		1.04(.69–1.55)	
Greater Accra	.70(.49–1.02)		1.10(.71–1.73)	
Volta	1.07(.72–1.57)		.88(.57–1.35)	
Eastern	.66(.45-.97)		.74(.48–1.13)	
Ashanti	.60(.42-.87)		.68(.46–1.10)	
Brong Ahafo	1.33(.87–2.03)		1.22(.79–1.88)	
Northern	3.33(.87–2.03)		2.16(1.39–1.34)	
Upper East	2.65(1.83–3.83)		1.88(1.18–2.98)	
Upper West	3.40(2.38–4.85)		2.72(1.75–4.22)	

## Discussion

Anaemia is a major public health problem in developing countries. Promoting access to healthcare services is crucial for anaemia prevention and management. Health insurance is a major intervention that promotes financial access to healthcare services. This study sought to examine the association between health insurance membership and anaemia status among Ghanaian children under-five years.

It was found that health insurance membership was significantly associated with anaemia status. Children who were not insured with the NHIS were more likely to be anaemic. This finding is supported by prior studies. For example, [16] found that insured children were about seven times less likely to be anaemic compared to non-insured children [27, 32]. The evidence implies that health insurance membership is a protective factor against anaemia among children. Unfortunately, many of the children were not insured. This finding suggests that mothers/caregivers of non-insured children may face financial barriers to accessing healthcare services for their children. It is also possible that mothers/caregivers of non-insured children may resort to unorthodox methods of care, such as herbal medication or delay in seeking medical attention. As a result, their children may develop complications, which can lead to disability or mortality. Even if they decide to seek medical attention, they would have to pay out of pocket. Consequently, they are being exposed to catastrophic healthcare expenditure which can lead poverty. Underlying reasons for the low enrolment on the NHIS may include mothers/caregivers inability to afford insurance premium, perceived poor quality of services, dissatisfaction with service delivery and delays in renewing NHIS membership [33–35]. Although the introduction of the mobile phone renewal system can help reduce delays in membership renewal, it cannot address barriers such as perceived poor quality of care. Going forward, management of the National Health Insurance Authority would have to devise innovative strategies to help increase enrolment among mothers/caregivers of children under-five years.

Another important finding from this study was the high prevalence of anaemia among children. Children under-two years, those of low socio-economic status, and those from northern Ghana were more likely to be anaemic. This is parallel with existing studies [6]. For example, [36], found that anaemia among children was more prevalent in the Upper East and Upper West Regions of Ghana. The authors also found that children below two years were more likely to be anaemic compared to children above two years. A cross-sectional survey on pediatric anaemia in rural Ghana also found that younger age was a risk factor of anaemia [37]. In Ethiopia, [2] revealed that children of low socio-economic status were five times more likely to be anaemic compared to children of high socio-economic status. These findings may be explained by poverty-related malnutrition [38]. It is evident that poor mothers/caregivers are less likely to have reliable access to sufficient quantity of nutritious food. In addition, the Ghana Living Standard Survey Round 7 report suggests that the Northern, Upper East and Upper West Regions are leading poor regions in Ghana [39]. These findings are also supported by prior studies [2, 40]. In this regard, poverty reduction should be considered as part of the strategies to eradicate anaemia in Ghana.

Furthermore, statistics suggest that the prevalence of anaemia among Ghanaian children under-five years has been fluctuating. For instance, according to the Ghana Demographic and Health Survey reports, the prevalence of anaemia among children under-five increased from 76% to 78% between 2003 and 2008 [41, 42]. Comparatively, the prevalence rate found in this study (MICS 2011) (57%) is lower than the prevalence rate found in the 2014 Ghana Demographic and Health Survey [43]. This suggests an increase in anaemia prevalence. The evidence implies that anaemia among children under-five years remain a public health concern in Ghana. More importantly, the current trend suggests that interventions aimed at eradicating pediatric anaemia seem to be making little progress.

Going forward, there is the need for stakeholders to reinforce anaemia prevention interventions, such as educating mothers/caregivers about recommended breastfeeding and child feeding practices. Above all, stakeholders must focus on eliminating demand-side barriers to accessing child healthcare services including cost. Since timely access to healthcare is crucial for early diagnosis and effective treatment outcomes, it is recommended that child healthcare services should be free of charge, since high cost of care is major barriers to healthcare utilization. Although children under-years are exempted from paying health insurance premiums in Ghana, they can only access free healthcare if their mothers’/caregivers’ have insured with the NHIS. In the quest to eradicate anaemia among children, it would be necessary for stakeholders to review the current benefit package of the NHIS. All children under-five years should have access to free healthcare, irrespective of their mothers’ NHIS membership status. In addition, anaemia prevention interventions should focus on more vulnerable children, such as children from northern Ghana and children of low socio-economic status.

## Conclusions

Anaemia is prevalent among Ghanaian children under-five years. Health insurance is a protective factor against pediatric anaemia. Unfortunately, health insurance coverage among children in Ghana is low. In the quest to eradicate anaemia among Ghanaian children, it would be necessary for stakeholders to review the benefit package of NHIS coupled with prioritizing anaemia prevention interventions among more vulnerable children. This study provides invaluable information for child healthcare policies and health insurance reforms in Ghana. Notwithstanding, this study is not devoid of limitations. One limitation is the quantitative nature of the study. Therefore, qualitative studies are required to expose the many intricate views of mothers/caregivers relating to issues of low enrolment on the National Health Insurance Scheme.
